# Modeling variation in mixture effects over space with a Bayesian spatially varying mixture model

**DOI:** 10.1002/sim.10022

**Published:** 2024-02-02

**Authors:** Joseph Boyle, Mary H. Ward, James R. Cerhan, Nat Rothman, David C. Wheeler

**Affiliations:** 1 Department of Biostatistics, Virginia Commonwealth University, Richmond, Virginia, USA; 2 Occupational and Environmental Epidemiology Branch, Division of Cancer Epidemiology and Genetics, National Cancer Institute, Rockville, Maryland, USA; 3 Department of Quantitative Health Sciences, Mayo Clinic, Rochester, Minnesota, USA

**Keywords:** Bayesian, case-control study, chemical mixtures, non-Hodgkin lymphoma, pesticides, polychlorinated biphenyls, polycyclic aromatic hydrocarbons, spatial statistics

## Abstract

Mixture analysis is an emerging statistical tool in epidemiological research that seeks to estimate the health effects associated with mixtures of several exposures. This approach acknowledges that individuals experience many simultaneous exposures and it can estimate the relative importance of components in the mixture. Health effects due to mixtures may vary over space driven by to political, demographic, environmental, or other differences. In such cases, estimating a global mixture effect without accounting for spatial variation would induce bias in effect estimates and potentially lower statistical power. To date, no methods have been developed to estimate spatially varying chemical mixture effects. We developed a Bayesian spatially varying mixture model that estimates spatially varying mixture effects and the importance weights of components in the mixture, while adjusting for covariates. We demonstrate the efficacy of the model through a simulation study that varies the number of mixtures (one and two) and spatial pattern (global, one-dimensional, radial) and magnitude of mixture effects, showing that the model is able to accurately reproduce the spatial pattern of mixture effects across a diverse set of scenarios. Finally, we apply our model to a multi-center case-control study of non-Hodgkin lymphoma (NHL) in Detroit, Iowa, Los Angeles, and Seattle. We identify significant spatially varying positive and inverse associations with NHL for two mixtures of pesticides in Iowa and do not find strong spatial effects at the other three centers. In conclusion, the Bayesian spatially varying mixture model represents a novel method for modeling spatial variation in mixture effects.

## 1 | INTRODUCTION

A common objective in epidemiology is to estimate the effects of one or many groups of related variables on a health outcome of interest. This approach, known as mixture analysis, is advantageous compared to single-variable analysis because it allows for the estimation of simultaneous effects of variables and enables the interpretation of estimates as across all other variables in the model. This also allows comparison of importance between variables. Mixture analysis supports the idea of studying the exposome, which is a concept embodying “every exposure to which an individual is subjected from conception to death”.^1^ The components of interest in these mixtures, or groups, are generally chemicals,^2–6^ but are sometimes socio-economic variables.^7–9^

One frequent complication in mixture analysis is that components in the mixture are commonly correlated. For example, an analysis of chemicals measured in house dust for their association with non-Hodgkin lymphoma (NHL) found many high correlations (>0.80) between several different polycyclic aromatic hydrocarbons (PAHs) and between several different congeners of polychlorinated biphenyls (PCBs).^10^ Such high correlations challenge traditional regression methods, inducing inflated standard errors in and potentially changing the signs of estimates, in a phenomenon known as the reversal paradox.^11^ Therefore, methods to accommodate these correlations are needed. Bayesian kernel machine regression models^12^ estimate a smooth kernel function separately for each relationship between mixture components and the outcome. Random forests predict the outcome by creating many sequences of decision rules over bootstrapped data samples.^13^ However, despite providing excellent predictive properties, they are unable to offer a coherent estimate of the effects of mixtures and their components. Additionally, quantile g-computation employs a causal inference framework to estimate the mixture effects that would be obtained from a hypothetical prospective randomized trial in which the treatment modified the quantiles of each mixture component.^14^ However, this technique relies on several structural assumptions (causal consistency, as well as a lack of both interference and unmeasured confounding) which may be violated in observational studies. Finally, the Bayesian group index model employs Bayesian estimation to estimate the effects of one or many mixtures, as well as the relative importance of components within the mixtures.^15^ This model represented an improvement upon its predecessor, weighted quantile sum (WQS) regression,^16^ by allowing the estimation of effects of multiple group indices potentially varying in direction and magnitude and by allowing estimation to occur in one step without data splitting. Through simulation, this model demonstrated an ability to accurately and consistently estimate group effects and to identify the important components of each group with good sensitivity and specificity.^15^

Mixture analysis may be further complicated by the possibility that the effect of the mixture is not constant over space. This scenario could occur if measured or unmeasured covariates in certain areas of a study region confound the association of the mixture, or if certain areas have very low or high values for many components in the mixture. This idea of nonstationarity in the mixture effect over space^17^ has taken hold in epidemiology,^10,18,19^ ecology,^20^ and economics.^21^ Notably, in the presence of spatial variation in the mixture effect, failing to incorporate it in the modeling framework could lead to biased effect estimates, because the estimation of a globally-constant effect would both over- and under-estimate the true effects in different areas. Additionally, failure to model existing spatial variation could result in reduced statistical power if estimation of a globally-constant and null effect obscured areas where the mixture effect was non-null.

Several methods have been developed to estimate spatially varying coefficients (SVC) in a regression framework. Geographically-weighted regression (GWR)^22^ models estimate a vector of regression coefficients at each location using a geographical weight matrix that assigns greater weight to observations at nearer locations and relies on a spatial bandwidth parameter that must be estimated. Despite the intuitive appeal of GWR, simulation studies have revealed extremely high correlations between sets of estimated parameters from the framework as well as biased estimates,^23^ and sub-optimal predictive ability.^24^ An improvement to GWR, the geographically-weighted LASSO (GWL)^25^ employed the shrinkage properties of LASSO regression for variable selection. In simulation studies, GWL performed better than GWR in prediction and effect estimation when the explanatory variables exhibited collinearity.^25^ However, moderate correlation in the resulting parameter estimates of GWL remained, and the smoothing of effect estimates somewhat obscured the boundaries between existing and null effects.^10^ In contrast to these methods, spatially varying coefficient process (SVCP)^21^ models utilize the Bayesian estimation paradigm to estimate spatially varying effects. SVCP models treat the response variable as a realization of a spatial (Gaussian) process in order to predict coefficient values at unobserved locations. The improved predictive ability and increased flexibility with respect to smoothing the effects of different variables in the SVCP model compared to the GWR framework was demonstrated in an analysis of crime data in Houston.^19^

Here, we develop and evaluate a Bayesian SVC mixture model that unifies the ideas of mixture analysis and spatially varying effects. This model estimates the effects of mixtures of correlated exposures, allowing the estimates to vary over space, and estimates the relative importance of components in the mixtures. We evaluate the efficacy of the proposed model through a simulation study and we apply the model to data from the National Cancer Institute (NCI) surveillance, epidemiology, and end results (SEER) case-control study of NHL to test for spatially varying effects of chemical mixtures and obtain more spatially precise regions for significant associations between chemical mixtures and NHL.

## 2 | METHODS

### 2.1 | Model specification

Assume a case-control setting, where for the *i*th subject, the binary outcome variable *Y_i_* has a Bernoulli distribution with probability parameter *p_i_*. Model the logit of the probability as $\log\left(\frac{p_{i}}{1-p_{i}}\right)=\beta_{i0}+\sum_{j=1}^{C}\beta_{ij}\sum_{k=1}^{C_{j}}\omega_{jk}q_{ijk}+\sum_{b=1}^{B}\theta_{b}x_{ib}$. Here $\beta_{i0}$ represents a spatially varying intercept term, and the set $\left[\beta_{i1},\ldots ,\beta_{iC}\right]$ are SVC (whose prior distributions are described in more detail below) that represent effects for the *C* mixtures in the model. The *j*th mixture has importance weights $\left[\omega_{j1},\ldots ,\omega_{jC_{j}}\right]$ which are subject to the constraints $\omega_{jk}\in \left(0,1\right)$ for all *k* and $\sum_{k=1}^{C_{j}}\omega_{jk}=1$ to aid in interpretability. The components in the mixture can be scored into quantiles (eg, quartiles or deciles) in order to reduce collinearity between components and accommodate different scales of measurement. Thus, the term $q_{ijk}$ represents for the *i*th subject the *k*th component in the *j*th mixture. Additionally, the vector $\theta =\left[\theta_{1},\ldots ,\theta_{B}\right]$ gives the (spatially-constant) coefficients for the adjustment covariates $\left[x_{i1},\ldots ,x_{iB}\right]$. We note that for other applications, the distribution of the outcome variable *Y_i_* need not be Bernoulli and could be normal for a continuous outcome or Poisson for a count outcome. Depending on the form of the outcome variable in question, the right-hand side of the model specification holds, and only the link function would change.

### 2.2 | Simulation study design

We created a simulation study that covered a variety of plausible scenarios to test the efficacy of the model in estimating spatially varying and spatially-constant mixture effects. The study region was defined to be the unit square of (0,1) × (0,1). The simulations used a case-control study design with an approximately 1:1 case to control ratio. Positive magnitudes for the mixture effects described below can be interpreted as increasing the likelihood of being a case.

The first class of scenarios involved one mixture effect. In the first (scenario 1A), the effect was globally normally distributed with mean 0 across the study region. This tested the ability of the model to reliably estimate a global or stationary effect without introducing artificial spatial patterning in mixture effect estimates. The second scenario (1B) consisted of a mixture effect that was normal with mean radially decreasing from 3 in the center of the study region to 0 on the boundaries of the region. This scenario tested the ability of the model to estimate spatially varying effects that vary smoothly over space, which could mimic an effect that decreases with increasing distance from a point source such as a chemical facility. The third scenario (1C) consisted of an effect that varied in one direction (horizontally over the unit square). In the left, middle, and right thirds of the study region, defined by the vertical boundary lines $x=\frac{1}{3}$ and $x=\frac{2}{3}$, the mixture effect was normally distributed with means 3, 1.5, and 0, respectively. This tested the ability of the model to estimate spatially varying effects in the presence of “invisible” boundaries, which could arise from policy differences in different administrative regions.

The second class of scenarios built upon the first class and involved two mixture effects. In the first (scenario 2A), one of the effects varied radially as in scenario 1B, and the other effect was globally normally distributed with mean 1. In the second (2B), one of the effects varied in one dimension as in scenario 1C and the other effect was globally normally distributed with mean 1. These scenarios tested the ability of the model to simultaneously estimate both spatially varying and spatially-constant mixture effects, all of which were generated with a small random perturbation via a SD of 0.1. Table 1 summarizes the different simulation scenarios. For each scenario, we generated D=50 datasets and fit models to each dataset.

The mixture components in the simulation scenarios were generated from correlated distributions to imitate natural patterns of chemical or socio-economic variables.^26^ In the first class of scenarios, the mixture consisted of $C_{1}=6$ components with importance weights $\omega_{1}=\left[0.30,0.20,0.20,0.13,0.10,0.07\right]$, and the component values were generated from a multivariate normal distribution with correlation matrix given by $\begin{array}{cccccc} 1 & 0.3 & 0.2 & 0.1 & 0 & 0\\ 0.3 & 1 & 0.2 & 0.1 & 0 & 0\\ 0.2 & 0.2 & 1 & 0.1 & 0 & 0\\ 0.1 & 0.1 & 0.1 & 1 & 0 & 0\\ 0 & 0 & 0 & 0 & 1 & 0\\ 0 & 0 & 0 & 0 & 0 & 1 \end{array}$. In the second class of scenarios, the first mixture was as above, and the second mixture consisted of $C_{2}=6$ components with importance weights $\omega_{2}=\left[0.45,0.15,0.15,0.15,0.05,0.05\right]$. The component values for the second mixture were generated from a multivariate normal distribution with correlation matrix $\begin{array}{cccccc} 1 & 0.2 & 0.1 & 0.1 & 0 & 0\\ 0.2 & 1 & 0.2 & 0.1 & 0 & 0\\ 0.1 & 0.2 & 1 & 0.1 & 0 & 0\\ 0.1 & 0.1 & 0.1 & 1 & 0 & 0\\ 0 & 0 & 0 & 0 & 1 & 0\\ 0 & 0 & 0 & 0 & 0 & 1 \end{array}$. The mean values varied for components in the mixtures but we omit them here for brevity because all components are quantized in the Bayesian index model (eg, *q* = 0, 1, 2, 3 for quartiles) to ensure common component scaling. For simplicity, the simulation study did not incorporate adjustment covariates.

### 2.3 | Model fitting

For each scenario, we fit a Bayesian SVC mixture model to each simulated dataset. We fit the models using Markov chain Monte Carlo (MCMC) methods and used the following prior distributions for parameters in the model. Each spatially varying mixture coefficient received the spatial prior $\beta_{j}∼\text{MVN}\left(0,\tau_{\beta_{j}}\Omega^{-1}\right)$, where MVN denotes the Multivariate Normal distribution. In this specification, the matrix Ω represents the spatial correlation between observations, with entry in the *j*th row and *k*th column (1+d(j,k)/ρ)×exp(−d(j,k)/ρ) giving the correlation between coefficients at the *j*th and *k*th locations with Euclidean distance d(j,k) between them. This correlation function comes from the Matern family, fixing parameters m=1 and $v=\frac{3}{2}$. We used the Matern family due to its popularity in the geostatistical literature,^27,28^ smoothness, and flexibility. We assigned a non-informative Dirichlet(α) prior to each vector of importance weights so that the weights would be non-negative and $\sum_{k=1}^{C_{j}}\omega_{jk}=1$ for the *j*th mixture. Additionally, the spatial range parameter ρ received a uniform prior over the range of inter-point distances.

We estimated model parameters using Just Another Gibbs Sampler (JAGS)^29^ in the software R, version 3.6.1.^30^ We used two chains in the MCMC simulations, burning in a large number of iterations depending on the scenario and sampling an appropriate (100 000-150 000) number of iterations from the approximate posterior distribution of parameters. In each iteration of the MCMC chain, we used the values of the spatially varying mixture estimates and the covariance function to predict the values over a fine 30 × 30 grid covering the study region. We monitored convergence of parameters using the Gelman-Rubin statistic,^31^ considering a parameter to have converged if its Gelman-Rubin statistic was below 1.1 using the coda package^32^ in R.

### 2.4 | Model evaluation

We evaluated model performance for each scenario in several ways. First, we calculated mean square error (MSE) between the true means of the mixture effects depending on their location and the mean of the posterior distribution of coefficient estimates for each observation. Second, we calculated MSE and median absolute error (MAE) between the true and estimated importance weights. We summarized these performance metrics over all generated datasets. Additionally, we mapped the average predicted grids of coefficients over the study region for each scenario and calculated the proportion of significant grid cells stratified by the appropriate components of the spatial process (eg, thirds of the study region for the one-dimensional scenarios). We considered a grid cell to be a location of a significant mixture effect using exceedance probabilities, which are the proportion of posterior samples that exceed the null value of zero and used a 95% threshold for the exceedance probabilities. Finally, for the multiple-group scenarios, we calculated the estimated correlation between the coefficient estimates.

### 2.5 | Application to NCI-SEER NHL study

The NCI-SEER NHL study is a multi-center population-based case-control study of NHL in four areas of the United States (Wayne, Oakland, and Macomb Counties, comprising the Detroit metropolitan region; Los Angeles County; King and Snohomish Counties, comprising the Seattle metropolitan region; and the state of Iowa). The study population, which has been described in detail previously,^33,34^ included 1321 NHL cases ages 20 to 74 years, diagnosed between July 1, 1998 and June 30, 2000, at one of the above four SEER registries. Population-based controls (N = 1057) were selected from each center using random-digit dialing for controls less than 65 years old and Medicare eligibility files for controls greater than or equal to 65 years. The controls were frequency matched to the cases by age (within 5-year groups), sex, and race. Cases and controls with a history of either NHL or HIV were excluded from the study. Participation rates were 76% and 52% for cases and controls, respectively. The study population in our analysis included participants who agreed to dust sampling (had at least half of their rugs and carpets for 5 years or more, 57%),^2^ had complete chemical measurements (described below), and had street-level geocoded addresses and key covariates recorded. Table 2 summaries the characteristics of this study population with dust samples by study center.

Study participants completed a lifetime residential history calendar, which asked them to state the complete address of any home they lived in, beginning from birth and including temporary or vacation homes where they lived for a total of at least 2 years. Interviewers completed in-person interviews with participants in which they reviewed the calendar with participants and attempted to resolve any discrepancies or missing data in the calendar. Residential addresses in the calendar were matched to databases of geographic addresses to obtain geographic coordinates.^35^ Interviewers took global positioning system (GPS) readings outside the home to obtain the coordinates for the current home.

The NCI-SEER NHL study measured chemical concentrations from samples of dust taken from vacuum cleaners of a subsample of consenting participants. The details of this process have been described previously.^2,36^ We analyzed four chemical mixtures in the Bayesian spatially varying mixture model: concentrations (ng/g dust) of PCBs (congeners 105, 138, 153, 170, 180); PAHs (Benz(a)anthracene, Benzo(a)pyrene, Benzo(b)fluoranthene, Benzo(k)fluoranthene, Chrysene, Dibenz(ah)anthracene, Indeno(1,2,3-cd)pyrene); Pesticides I (α-chlordane, γ-chlordane, carbaryl, dichlorodiphenyldichloroethylene (DDE), dichlorodiphenyltrichloroethane (DDT), *o*-phenylphenol, pentachlorophenol, propoxur); and pesticides II (chlorpyrifos, *cis*-permethrin, *trans*-permethrin, 2,4-D, diazinon, dicamba, methoxychlor). We chose this grouping of chemicals both due to chemical class (PCBs and PAHs) and owing to its use in previous chemical analyses of NHL risk based on univariate associations (positive and negative associations with NHL for Pesticides I and II, respectively).^10,37^ The distribution of chemical exposures was quite different for different study centers, reflecting differing prevalence of chemicals in different regions of the country (Table S1). For the data analysis we scored chemical concentrations into quartiles (*q* = 0, 1, 2, 3) for each variable.

We applied the Bayesian spatially varying mixture model to spatially model the association between each mixture of chemical exposures and NHL status at each study center, treating NHL status as a binary response variable *Y* taking values of 1 and 0 for cases and controls, respectively. We fitted models at each study center to allow for differences in chemical exposure profiles and strengths of association with NHL status in different regions of the country. We adjusted for age, gender (male vs reference female), race (Black or other vs reference White), and level of education (college degree or high school degree vs reference less than high school degree) in all models, as done in previous analyses of the NCI-SEER NHL study.^2,38,39^

We specified the priors as in the simulation study and assigned a normal prior $\theta_{i}∼N\left(0,\tau_{i}\right)$ where $\tau_{i}=\frac{1}{\sigma_{i}^{2}}$ and $\sigma_{i}∼\text{Unif}\left(0,10\right)$ for each adjustment covariate. We fitted models using a burn-in period of 100 000 iterations and retaining 20 000 for sampling from the joint posterior distribution. We performed inference on the chemical groups using exceedance probabilities, considering an association between chemical group and risk for NHL to be significantly positive (negative) if its exceedance probability of being elevated (lowered) was greater than 95%. We calculated the spatially varying odds ratio (OR) for the mixture effects by exponentiating the coefficients from the posterior samples. In addition, we mapped the coefficients for the mixture effects for participants at each study center in order to visualize trends in the spatially varying associations.

## 3 | RESULTS

### 3.1 | Simulation study

**FIGURE 1:**
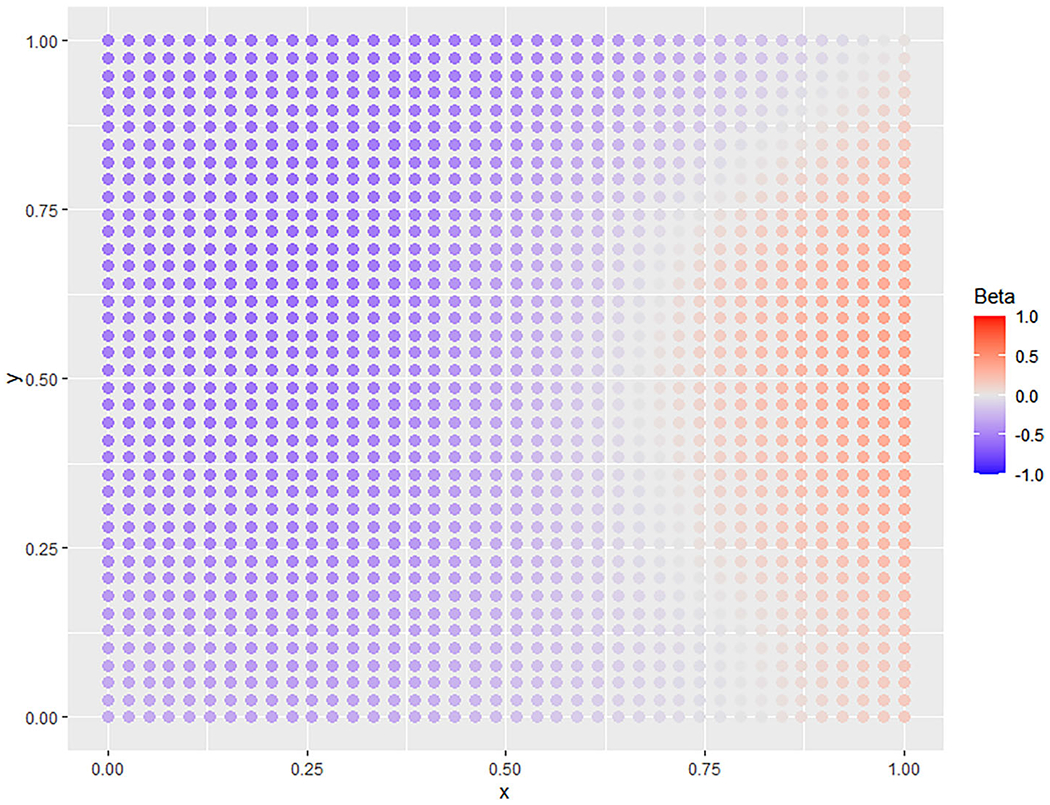
Average estimated coefficient surface for simulation study scenario 1A. The study region was the unit square (0,1) × (0,1).

The summary of mixture effect estimates from the simulation study demonstrates the ability of the Bayesian spatially varying mixture model to estimate mixture effects that vary or are constant over space (Table 3). In the one-mixture scenarios, the characteristics of the true spatial process tended to be recovered by estimates from the model. For example, in scenario 1A, where the true effect had a zero mean everywhere in the study region, only 3.1% of grid cells had values significantly different than zero. There was little spatial patterning evident in the map of grid cell predictions (Figure 1). The mean predicted values slightly increased with increasing values of the *x*-coordinate, but nearly all (96.9%) of the grid cells were correctly predicted not to have mixture effects significantly different than the true value of zero. Thus, the true global pattern of a null mixture effect was recovered by the model.

**FIGURE 2:**
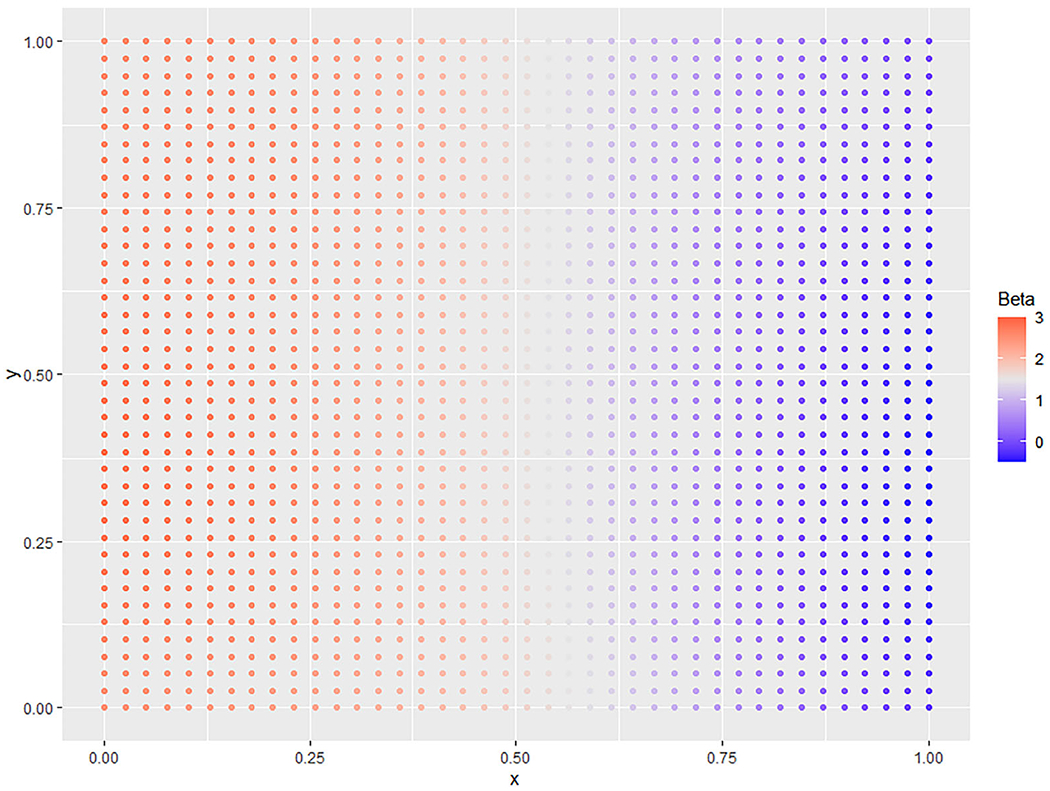
Average estimated coefficient surface for simulation study scenario 1B. The study region was the unit square (0,1) × (0,1).

In scenario 1B, where the mixture effect varied in one dimension, the proportion of significantly elevated grid cells was 99.5% where the true mean was 3, 81.7% where the true mean was 1.5, and 11.5% where the true mean was zero. These values demonstrate high power of the model to detect large and moderate spatially varying effects and reasonable ability to resist estimating false non-null effects when the true mixture effect is null. The delineation of the boundaries defining changes in means for the mixture effect was evident in the map of predicted grid cells (Figure 2), where each third of the study region moving from left to right corresponded to a sub-region of lower predicted effects.

**FIGURE 3:**
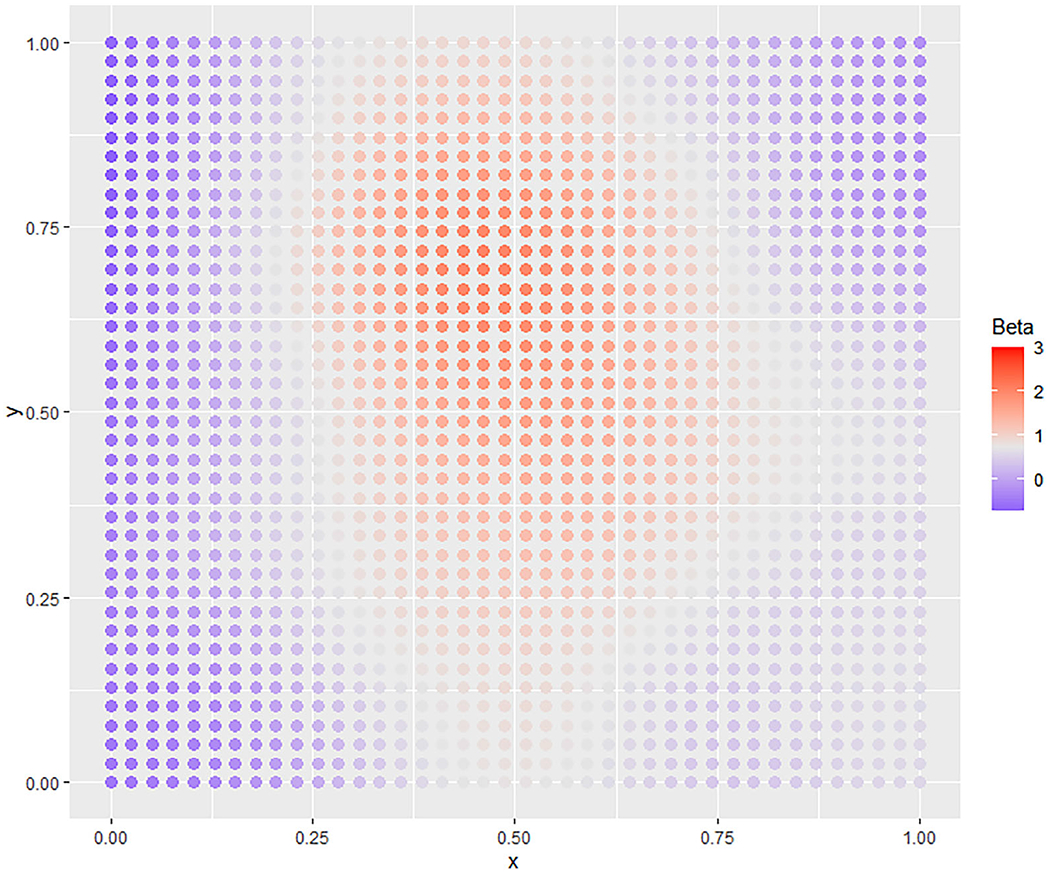
Average estimated coefficient surface for simulation study scenario 1C. The study region was the unit square (0,1) × (0,1).

These values suggest high power to detect moderate to large spatially varying effects in addition to partial recovery of the boundary separating a moderate effect from a null one. Finally, the mixture effect in scenario 1C that radially decreased from the center demonstrates similar statistical power for this spatial pattern. In subsets of the region where the true mixture effect mean was at least 2, 1, and 0.25, the proportion of significantly elevated grid cells are 95.5, 85.7, and 74.1, respectively, showing adequate power to detect smaller mixture effects. This process was captured with reasonable accuracy in the map of predicted grid cells (Figure 3). Finally, the MSE for the estimated coefficients was acceptable given their true magnitudes, and the accuracy of the estimated weights varied little for the different scenarios.

In the two-mixture scenarios, the model tended to capture the spatial variation in the first mixture effect while generally resisting inducing spatial patterning in the constant mixture effect (Table 3). In scenario 2A, the proportion of significantly elevated grid cells for the spatially varying mixture effect was 98.3% where the true mean was 3, 77.8% where the true mean was 1.5, and 9.5% where the true mean was zero. These quantities were slightly lower than their analogues for scenario 1B, suggesting that simultaneous estimation of a second mixture effect did not greatly hinder estimation of the spatially varying one.

**FIGURE 4:**
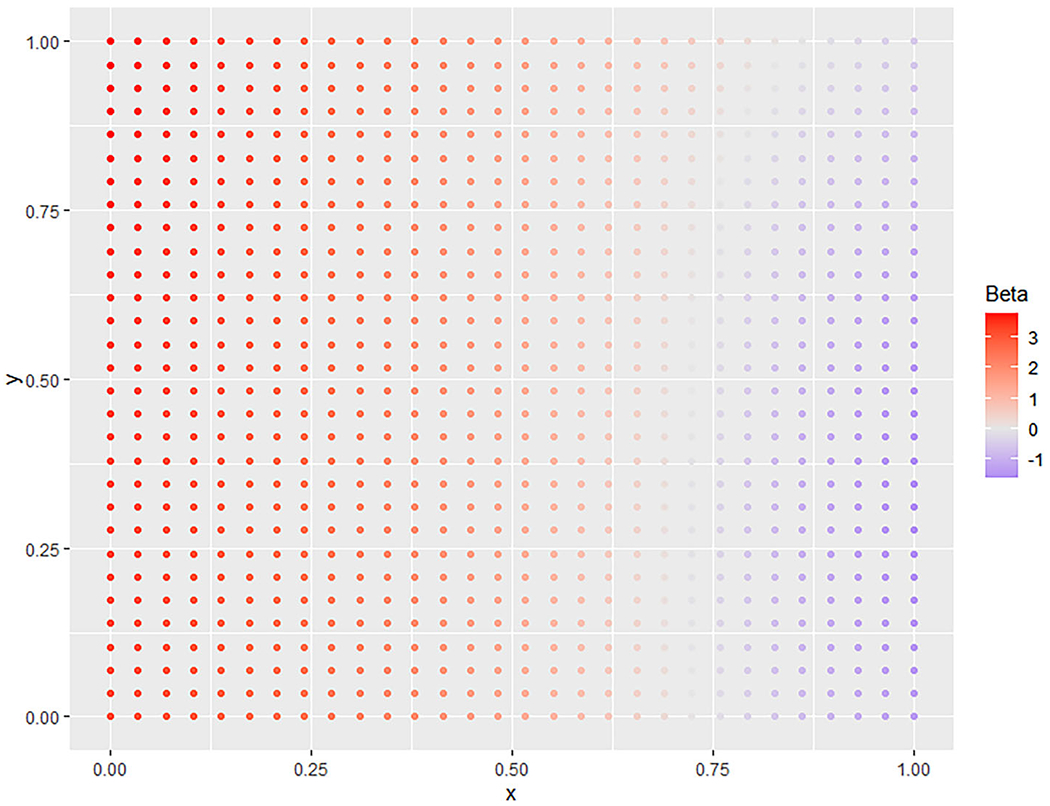
Average estimated coefficient surface for simulation study scenario 2A and spatially varying effect. The study region was the unit square (0,1) × (0,1).

**FIGURE 5:**
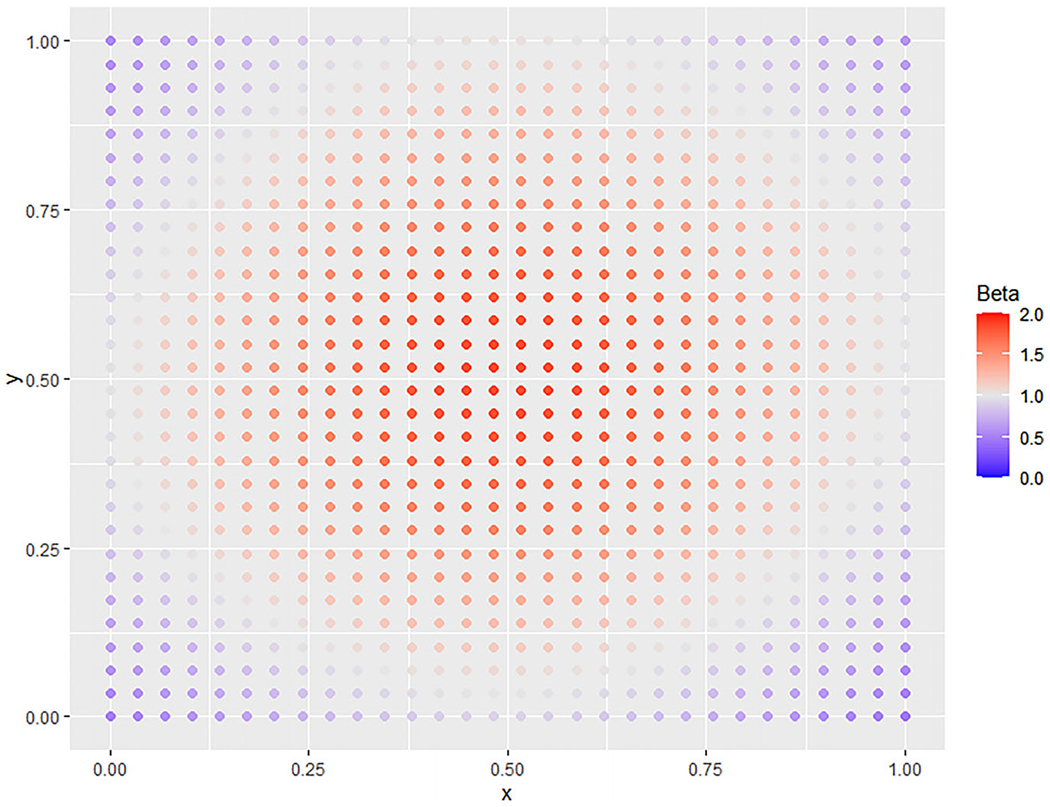
Average estimated coefficient surface for simulation study scenario 2B and spatially varying effect. The study region was the unit square (0,1) × (0,1).

The one-dimensional nature of the first mixture effect was recovered in the map of predicted grid cells (Figure 4), and though there was a slight increase in values of predicted grid cells for the spatially-constant effect from the northeast to southwest of the study region (Figure S1), the magnitude of this variation was small. In scenario 2B, the proportion of significantly elevated grid cells for the spatially varying mixture effect was 93.4 where the true mean was at least 2, 81.2 where the true mean was at least 1, and 20.3 where the true mean was at least 0.25. These values were very similar to the one-mixture scenario 1C for the two most central subsets of the study region (means of at least 2 and 1), and lower for the outer subset (mean of at least 0.25). The radial decrease of the mixture effect from the center of the study region was recovered by the model (Figure 5), and the spatially constant mixture effect was recovered in pattern and magnitude (Figure S2).

### 3.2 | Application to the NCI-SEER NHL study

**FIGURE 6:**
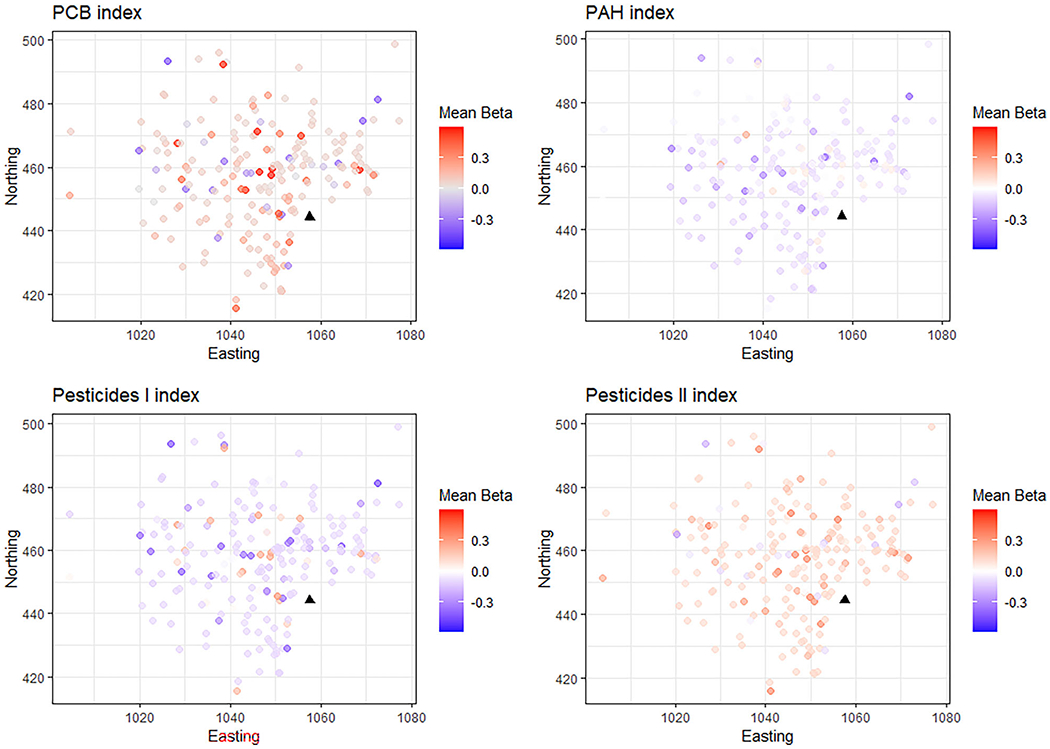
Summary of spatially varying chemical mixture associations in Detroit from the NCI-SEER NHL study application. PCBs and PAHs stand for polychlorinated biphenyls and polycyclic aromatic hydrocarbons, respectively. All points have been slightly jittered to maintain privacy. The city center is displayed with a black triangle.

In Detroit, the PCBs mixture had the greatest association with NHL risk among the four mixtures analyzed (Figure 6). Different subregions at this study center experienced different spatially varying associations of this mixture with NHL risk. In a region northwest of the city center, near the center of the map, several participants had high estimated coefficients for the PCB mixture of between 0.40 and 0.60 (corresponding to odds ratios of 1.49-1.82). In other regions in Detroit, the estimated mixture effect was closer to the null, and participants further from the city center tended to have negative mixture effect estimates of between −0.30 and −0.40 (corresponding to odds ratios of 0.74-0.67). In comparison, the magnitude of the associations between the PAHs, Pesticides I, and Pesticides II indices and NHL were much smaller. The spatial pattern of the parameter estimates for the Pesticides I mixture was similar to that of the PCBs mixture, with participants slightly northwest of the city center having positive mixture estimates and those far from the city center having negative mixture estimates, but more attenuated. In the Pesticides II mixture, *cis*-permethrin (0.163) and *trans*-permethrin (0.153) received the largest importance weights (Table S2).

**FIGURE 7:**
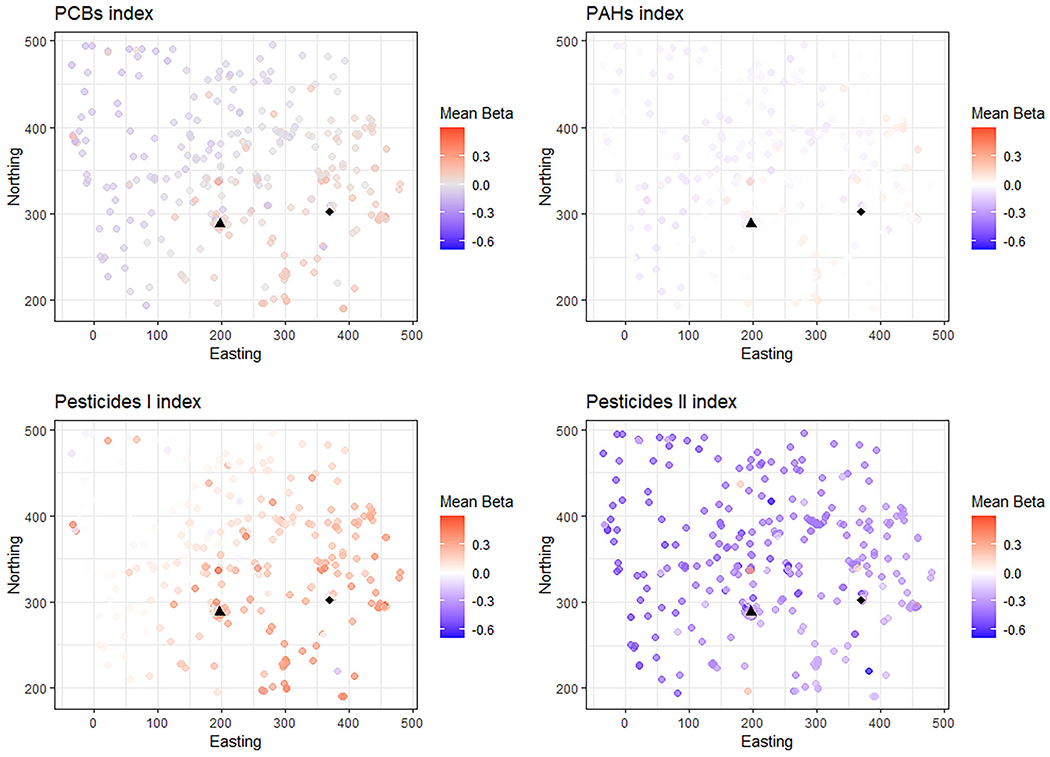
Summary of spatially varying chemical mixture associations in Iowa from the NCI-SEER NHL study application. PCBs and PAHs stand for polychlorinated biphenyls and polycyclic aromatic hydrocarbons, respectively. All points have been slightly jittered to maintain privacy. Des Moines and Iowa City are displayed with a black triangle and diamond, respectively.

In Iowa, the Pesticides I mixture had the greatest positive association with NHL risk (Figure 7). The southeast region of the state south of Des Moines and Iowa City had many estimated coefficients for this mixture of between 0.26 and 0.41 (corresponding to odds ratios of 1.30-1.50). The mixture effect was significant for five participants using 95% exceedance probabilities and for 50 participants using 90% exceedance probabilities (significance maps shown in Figure S3), and the three chemicals with the largest estimated importance weights in the mixture were propoxur (0.167), DDE (0.169), and DDT (0.124) (Table S1). All of the five participants with a significant mixture effect using 95% exceedance probabilities were cases, and 33 of the 50 participants with a significant mixture effect using 90% exceedance probabilities were cases. Meanwhile, the Pesticides II mixture had negative estimated coefficients for almost all of the study region, with the largest of these occurring in the north-central part of the state. These coefficients ranged from −0.35 to −0.60 (corresponding to odds ratios of 0.70-0.55) for the 162 participants with significant negative Pesticides II mixture effects using 95% exceedance probabilities (significance maps shown in Figure S4). The effect for this mixture of pesticides was significant for 249 participants using 90% exceedance probabilities. The chemical with the largest importance weight in this mixture was 2,4-D (0.390). Compared to these mixtures, PCBs had associations close to the null, with a general trend of small positive estimated coefficients in the south and southeast portions of the state and small negative estimated coefficients in the north and northwest portions of the state. The spatial pattern for PAH mixture associations was similar to that of PCBs but even more attenuated in magnitude.

**FIGURE 8:**
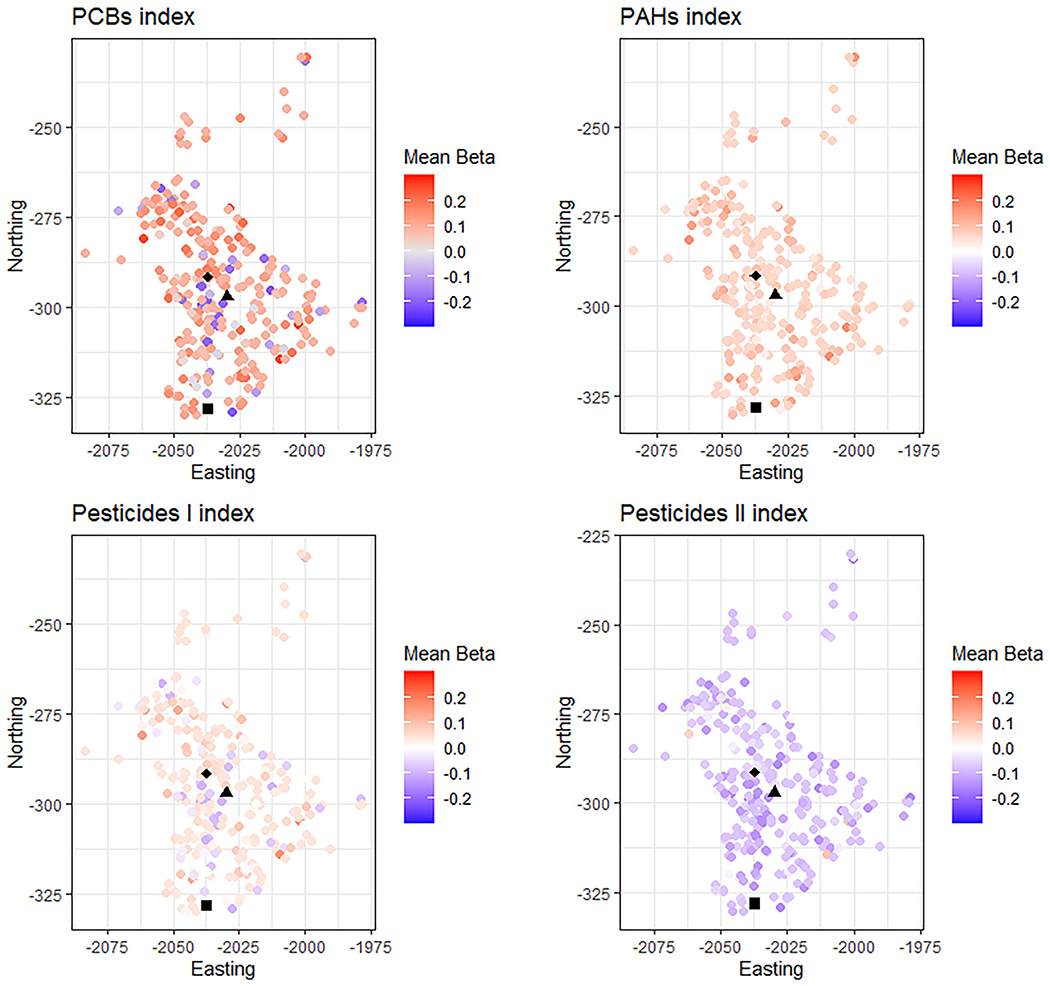
Summary of spatially varying chemical mixture associations in Los Angeles from the NCI-SEER NHL study application. PCBs and PAHs stand for polychlorinated biphenyls and polycyclic aromatic hydrocarbons, respectively. All points have been slightly jittered to maintain privacy The city center, Hollywood, and Long Beach are displayed with a black triangle, diamond, and square, respectively.

Maps of the spatially varying mixture coefficients in Los Angeles show the stronger associations of PCBs with NHL risk at this study center than of the other three chemical mixtures (Figure 8). The mean coefficient for the mixture of the PCBs varied over the study region but was often between 0.1 and 0.3 (corresponding to odds ratios of between 1.10 and 1.35), with the largest mean values in the southeast of the study region near Long Beach and in the northwest near Hollywood. Additionally, there was moderate to high spatial clustering of the PCB mixture coefficients, with several groups of positive coefficients for proximate study participants. Closer to the city center there was a smaller cluster of negative coefficients for the PCB mixture. Compared to PCBs, the associations with the PAHs and Pesticides I mixture were closer to the null. The PAH mixture had small and positive coefficients over the entire study region, whereas the spatial pattern of the Pesticides I mixture was similar to that of the PCBs mixture with smaller coefficients. Finally, the Pesticides II mixture had an inverse association with NHL risk for much of the study center, with most negative coefficients occurring near the city center.

**FIGURE 9:**
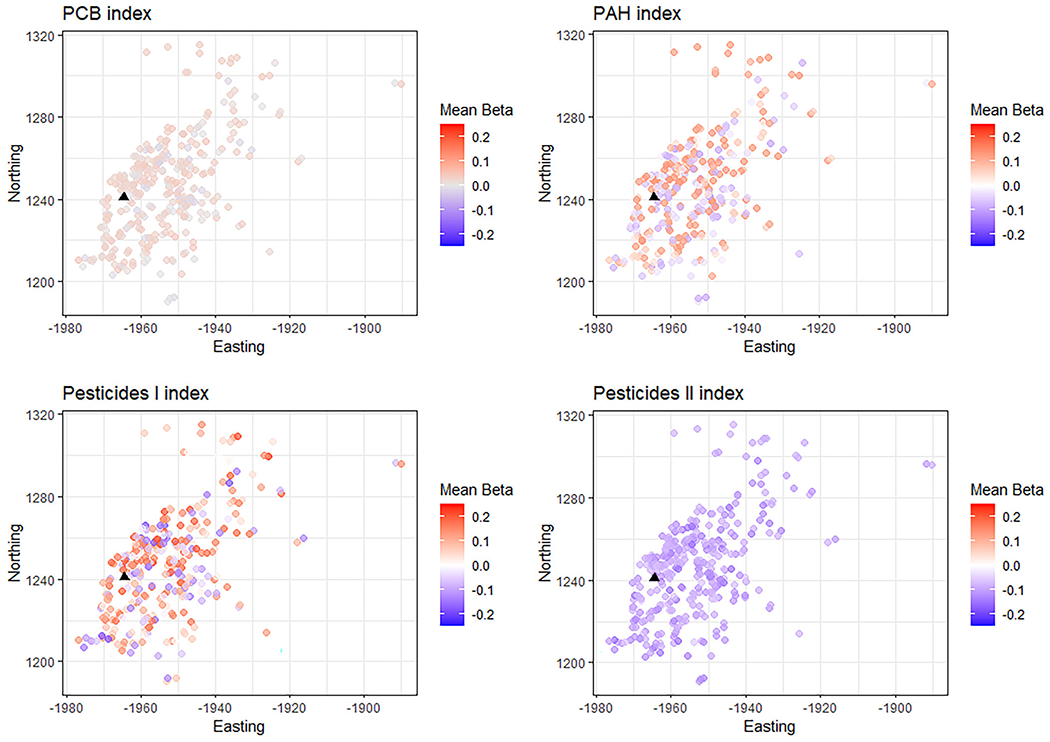
Summary of spatially varying chemical mixture associations in Seattle from the NCI-SEER NHL study application. PCBs and PAHs stand for polychlorinated biphenyls and polycyclic aromatic hydrocarbons, respectively. All points have been slightly jittered to maintain privacy. The city center is displayed with a black triangle.

In Seattle, the Pesticides II mixture had the most apparent spatial patterning, having negative estimated coefficients for the entire study center with the smallest coefficients generally located in the city center (Figure 9). In this mixture, Dicamba (0.171), Methoxychlor (0.152) had the largest importance weights. The mixture of PAHs tended to have positive estimated coefficients for participants across the study center, with some further from the city center having negative coefficients. Meanwhile, the PCBs mixture had estimated coefficients close to the null value for all participants, and the Pesticides I mixture had null to slightly positive coefficients.

## 4 | DISCUSSION

We developed the Bayesian spatially varying mixture model to estimate the effects of mixtures of variables that may vary over a study region. Although we designed the model for use in a case-control study to assess the effects of mixtures of chemicals, any mixture of variables such as socio-economic measures could be used and the outcome variable could be modified to a continuous or count data only by changing the link function. Thus, the Bayesian spatially varying mixture model can apply to a wide variety of modeling scenarios. We evaluated the performance of our model with a simulation study that contained several plausible patterns of spatial variation in mixture effects—null, one-dimensional, and radially decreasing variation—and varying number of mixtures having an association with the outcome. The components of each mixture were generated from a correlated distribution that could challenge several commonly used statistical methods such as ordinary least squares regression which assumes independence between components. In general, we found that our model was able to identify the spatial pattern in the mixture estimates regardless of the type of variation or whether there were one or two mixtures in the model. Notably, the model was able to identify spatial variation in mixture effects at unobserved locations as well as boundaries defining changes in mixture effects, and also estimate the importance weights of components in the mixture. The model we propose here is novel because it is the first to estimate spatially varying mixture effects rather than SVC or global mixture effects. Therefore, this model unifies spatial analysis and the exposome ideal by estimating how mixtures of many potentially correlated components have effects that vary over a spatial region.

We applied our spatially varying mixture model to the NCI-SEER NHL study that was conducted in four SEER centers in the United States and assessed the associations with concentrations of four mixtures of chemical exposures (PCBs, PAHs, and two groups of pesticides) with risk for NHL at each study center. Notably, we identified a positive and significant spatially varying association for a mixture of pesticides with NHL risk in eastern and southern Iowa, with propoxur, DDE, and DDT being the most important chemicals in this mixture. Of these chemicals, DDT has been identified by IARC as a probable carcinogen with limited evidence of carcinogenicity for NHL,^40^ though a previous study that performed single-chemical analyses found no association of DDE and DDT with NHL.^4^ Similarly, we found a negative and significant spatially varying association for another mixture of pesticides and NHL in northern and western Iowa, with 2,4-D being the most important chemical in this mixture. Residential use of this chemical has been identified as having a null association with NHL in previous analyses by itself in this dataset^41^ and in a mixture in a pooled analysis of several cohorts.^42^ The former result supports a previous Bayesian analysis of these data that estimated global mixture effects and cumulative unmeasured spatial risk simultaneously^43^ as well as one using WQS regression that found large importance weights for these and two other pesticides (γ-chlordane and *o*-phenylphenol) for a positive and significant mixture effect in Iowa.^10^ The latter result was also identified in the Bayesian analysis,^43^ and in another analysis a WQS index constrained to have positive association with NHL risk estimated a very small importance weight for 2,4-D, which is consistent with having a large weight in a mixture showing an inverse association with NHL.^44^ A primary contribution of our analysis in the context of these preceding analyses is that by allowing spatial variation in the mixture effects, the model allows increased spatial precision in determining which areas are the strongest drivers of a significant global effect estimate. This can motivate a public health or research response. For example, to address or further analyze the relationship between pesticides and NHL, it could be helpful to focus attention on southern and eastern Iowa, where pesticide use patterns may have varied from other regions of the state. There were other geographic differences in the distribution of chemical exposures in house dust (Table S1). In addition to participants from Iowa and Los Angeles tending to have the largest measurements for chemicals in the Pesticides I and II mixtures, those from Detroit often had the largest measurements for several PAHs. The geographic distribution of PCB congeners was similar between study centers.

Across the four study centers in our analysis, we identified several consistent if not significant associations between chemical mixtures and NHL risk. For example, the Pesticides I and Pesticides II mixtures tended to have positive and negative estimated coefficients respectively for participants in three of the four study centers, with the exceptions being Seattle for Pesticides I and Detroit for Pesticides II. Examining the geographic distributions of chemicals in these mixtures can provide insight into the one center that did not exhibit similar trends for a mixture as did the other three. Notably, exposures to Pesticides I chemicals were relatively low in Seattle and particularly for propoxur and DDT which received large importance weights for the significant associations in Iowa. Also, exposures to chemicals in the Pesticides II mixture were often lower in Detroit than in Iowa. Additionally, the PCBs mixture was generally positively associated with NHL risk in our analysis, and the mixture of PAHs had mostly null associations. In this way, our analysis allowed comparison of spatially varying associations in mixture effects within and across study centers. We chose to focus on estimating these spatially varying mixture associations in this study in the absence of spatial random effects, because identifiability would become a concern when estimating multiple spatially varying quantities simultaneously.^45^

The findings of our simulation study and data application suggest that the Bayesian spatially varying mixture model can provide an effective tool for researchers to estimate mixture effects that may vary over space. The model contributes to the literature by moving from estimating SVCs to estimating spatially varying mixture effects, which can address the simultaneous effects of many variables on an outcome in a hierarchical manner that also estimates the importance weights of each component of the mixture. A primary benefit of the model is that it borrows strength from neighboring observations in the data to stabilize mixture effect estimates and allow for proximate study participants to share similar effect estimates. This choice reflects the reasonable hypothesis that participants who are close to each other are more similar than those who are farther away, particularly with regard to chemical mixture exposures. Additionally, our model provides the full posterior distribution of all parameters of interest, which allows interpretation of estimates and inference to occur through summaries such as credible intervals and exceedance probabilities. Also, researchers can incorporate prior knowledge of mixture effect strengths or ranges of spatial correlation into the prior distributions. Finally, the model is able to control for additional covariates that may be associated with the outcome of interest.

It is important to discuss the strengths of the model in the context of its limitations. First, though the model allows for spatial variation in the mixture effect, it does not allow for such variation in the importance weights. It is possible that different components in the mixture could have different contributions to the overall mixture effect over space, and estimation of a global set of importance weights would not reflect this variation. It is less clear how to model spatial variation in a constrained set of weights that sum to one, and modeling spatial variation in the weights would further increase the computational cost in model fitting, but this limitation provides motivation for future research. Second, the model does not provide a means to test directly for spatial variation during model fitting. It is possible that the effect of some mixtures may truly be global over space. In this case, modeling spatial variation in the mixture effect would be unnecessary and even possibly inappropriate and would increase the computational time without need. Future work could test for the presence or absence of spatial variation in the mixture effect during model fitting, perhaps modeling the mixture effect itself as a statistical mixture of global and spatial distributions, in order to model globally constant effects more accurately. In addition, in the data application to the NCI-SEER NHL study, we controlled for a temporally-fixed number of adjustment covariates and modeled chemical mixtures that were measured at one time point. It is possible that in this or other studies, the effects of the mixtures or covariates could be time varying, which provides an opportunity to extend our model for such time varying effects. In addition, our application only used participants in the NCI-SEER study who had chemical exposures measured in house dust in their homes. This was a subset of the larger sample from the NCI-SEER study, and a previous analysis found that participants with dust measurements were more likely to be elderly, living in single-family homes, and have lived in their current home for longer.^2^ It is possible that some of the estimates or conclusions of spatial variation in mixture effect estimates could change if all study participants contributed dust samples. Finally, we analyzed all cases of NHL together, though there is some evidence of heterogeneity in the etiology of different NHL subtypes.^34^

## 5 | CONCLUSION

In conclusion, our Bayesian spatially varying mixture model represents a novel way to estimate effects in mixtures of variables that may vary over space. This method acts on mixtures of measured components and can be modified to accommodate a variety of outcome variables and mixture types. Therefore, it provides a broadly applicable tool for analysts in a wide range of fields. Outputs from the model are intuitive and are able to drive responses such as policy interventions. For example, a clustering of significant positive associations of a mixture of chemicals with disease risk can motivate subsequent spatially-precise investigations into the sources of elevated chemical exposures or inform the geographic area to target public health responses.

## Supplementary Material

Supplemental_Material

## Figures and Tables

**TABLE 1:** Summary of simulation scenarios for one and two mixtures with varying magnitude and spatial patterns.

Scenario number	Number of mixtures	Mixture description	Mixture magnitude and spatial pattern
1A	1	Constant	Mean of 0 globally

1B	1	One dimension	Means of 3, 1.5, and 0 in left, middle, and right thirds of study region

1C	1	Radial	Mean decreasing from 3 in center of study region to 0 on boundaries

2A	2	One dimension	Means of 3, 1.5, and 0 in left, middle, and right thirds of study region
		Constant	Mean of 1 globally

2B	2	Radial	Mean decreasing from 3 in center of study region to 0 on boundaries
		Constant	Mean of 1 globally

**TABLE 2:** Characteristics of the NCI-SEER NHL study population with house dust measurements by study center.

Center	Detroit	Iowa	Los Angeles	Seattle
Sample size	201	335	292	342

Status				
Case	127 (63)	188 (56)	168 (58)	182 (53)
Control	74 (37)	147 (44)	124 (42)	160 (47)
Age (years)	58 (11.4)	61 (11.2)	59 (11.2)	59 (10.8)

Sex				
Male	114 (57)	177 (53)	165 (57)	171 (50)
Female	87 (43)	158 (47)	127 (43)	171 (50)

Race				
White	164 (81)	331 (99)	215 (74)	316 (92)
Non-white	37 (19)	4 (1)	77 (26)	26 (8)

Education				
<12 years	23 (11)	32 (10)	31 (11)	19 (6)
12-15 years	124 (62)	241 (72)	171 (59)	201 (59)
≥16 years	54 (27)	62 (19)	90 (31)	122 (35)

*Note*: Age is summarized using mean (SD) and all other variables are summarized using count (percent). Some percentages may not sum exactly to one due to rounding.

**TABLE 3:** Summary of mixture effect estimates in the simulation study scenarios for one and two mixtures.

	One mixture			Two mixtures			
Metric	1A: Constant	1B: One Dimension	1C: Radial	2A: One Dimension	2A: Constant	2B: Radial	2B: Constant
Coefficient MSE	0.195	0.449	0.345	0.438	0.558	0.338	0.374

Weights MSE	0.007	0.012	0.011	0.009	0.018	0.007	0.014

Weights MAE	0.068	0.077	0.074	0.070	0.096	0.061	0.086

Correlation	-	-	-	0.438		0.504	

Significant grid cells							
Proportion	Mean of 0:0.031	Mean of 3:0.995	Mean of 2:0.955	Mean of 3:0.983	Mean of 1:0.147	Mean of 2:0.934	Mean of 1:0.203
	-	Mean of 1.5:0.817	Mean of 1:0.857	Mean of 1.5:0.778	-	Mean of 1:0.812	-
	-	Mean of 0:0.115	Mean of 0.25:0.741	Mean of 0:0.095	-	Mean of 0.25:0.203	-

*Note*: MSE and MAE refer to mean square error and mean absolute error, respectively. Quantities presented in table are the mean over all datasets in a scenario for importance weights MSE and MAE, correlation, and significant grid cells, and median for Coefficient MSE.

## Data Availability

The data that support the findings of this study are available on request from the study principal investigator Dr. Nat Rothman. The geospatial data are not publicly available due to privacy or ethical restrictions.

## References

[1] Wild CP. The exposome: from concept to utility. Int J Epidemiol. 2012;41(1):24–32. 22296988 10.1093/ije/dyr236

[2] Colt JS, Severson RK, Lubin J, Organochlorines in carpet dust and non-Hodgkin lymphoma. Epidemiology. 2005;16:516–525. 15951670 10.1097/01.ede.0000164811.25760.f1

[3] Brown LM, Blair A, Gibson R, Pesticide exposures and other agricultural risk factors for leukemia among men in Iowa and Minnesota. Cancer Res. 1990;50(20):6585–6591. 2208120

[4] De Roos AJ, Hartge P, Lubin HJ, Persistent organochlorine chemicals in plasma and risk of non-Hodgkin’s lymphoma. Cancer Res. 2005;65(23):11214–11226. 16322272 10.1158/0008-5472.CAN-05-1755

[5] Ward MH, Colt JS, Metayer C, Residential exposure to polychlorinated biphenyls and organochlorine pesticides and risk of childhood leukemia. Environ Health Perspect. 2009;117(6):1007–1013. 19590698 10.1289/ehp.0900583 PMC2702395

[6] Purdue MP, Hoppin JA, Blair A, Dosemeci M, Alavanja MCR. Occupational exposure to organochlorine insecticides and cancer incidence in the Agricultural Health Study. Int J Cancer. 2007;120(3):642–649. 17096337 10.1002/ijc.22258 PMC1971137

[7] Li Z, Christensen GM, Lah JJ, Neighborhood characteristics as confounders and effect modifiers for the association between air pollution exposure and subjective cognitive functioning. Environ Res. 2022;212:113221. 35378125 10.1016/j.envres.2022.113221 PMC9233127

[8] Wheeler DC, Boyle J, Barsell DJ, Associations of alcohol and tobacco retail outlet rates with neighborhood disadvantage. Int J Environ Res Public Health. 2022;19(3):1134. 35162162 10.3390/ijerph19031134 PMC8834944

[9] Lian M Geographic variation in maternal smoking during pregnancy in the Missouri Adolescent Female Twin Study (MOAFTS). PLoS One. 2016;11(4):e0153930. 10.1371/journal.pone.0153930 27100091 PMC4839577

[10] Czarnota J, Gennings C, Colt JS, Analysis of environmental chemical mixtures and non-Hodgkin lymphoma risk in the NCI-SEER NHL study. Environ Health Perspect. 2015;123(10):965–970. 25748701 10.1289/ehp.1408630 PMC4590749

[11] Tu Y-K, Gunnell D, Gilthorpe MS. Simpson’s paradox, Lord’s paradox, and suppression effects are the same phenomenon—the reversal paradox. Emerg Themes Epidemiol. 2008;5(1):1–9. 18211676 10.1186/1742-7622-5-2 PMC2254615

[12] Kim SS, Meeker JD, Keil AP, Exposure to 17 trace metals in pregnancy and associations with urinary oxidative stress biomarkers. Environ Res. 2019;179:108854. 31678726 10.1016/j.envres.2019.108854 PMC6907890

[13] Breiman L. Random forests. Mach Learn. 2001;45(1):5–32.

[14] Keil AP, Buckley JP, O’Brien KM, Ferguson KK, Zhao S, White AJ. A quantile-based g-computation approach to addressing the effects of exposure mixtures. Environ Health Perspect. 2020;128(4):47004. 32255670 10.1289/EHP5838 PMC7228100

[15] Wheeler DC, Rustom S, Carli M, Whitehead TP, Ward MH, Metayer C. Bayesian group index regression for modeling chemical mixtures and cancer risk. Int J Environ Res Public Health. 2021;18(7):3486. 33801661 10.3390/ijerph18073486 PMC8037139

[16] Carrico C, Gennings C, Wheeler DC, Factor-Litvak P. Characterization of weighted quantile sum regression for highly correlated data in a risk analysis setting. J Agric Biol Environ Stat. 2015;20(1):100–120. doi:10.1007/s13253-014-0180-3 30505142 PMC6261506

[17] Rollinson CR, Finley AO, Alexander MR, Working across space and time: nonstationarity in ecological research and application. Front Ecol Environ. 2021;19(1):66–72.

[18] Yao N, Foltz SM, Odisho AY, Wheeler DC. Geographic analysis of urologist density and prostate cancer mortality in the United States. PloS One. 2015;10(6):e0131578. 26110832 10.1371/journal.pone.0131578 PMC4482500

[19] Waller LA, Zhu L, Gotway CA, Gorman DM, Gruenewald PJ. Quantifying geographic variations in associations between alcohol distribution and violence: a comparison of geographically weighted regression and spatially varying coefficient models. Stoch Environ Res Risk Assess. 2007;21(5):573–588.

[20] Finley AO. Comparing spatially-varying coefficients models for analysis of ecological data with non-stationary and anisotropic residual dependence. Methods Ecol Evol. 2011;2(2):143–154.

[21] Gelfand AE, Kim H-J, Sirmans CF, Banerjee S. Spatial modeling with spatially varying coefficient processes. J Am Stat Assoc. 2003;98(462):387–396. 10.1198/016214503000170 PMC11484471 39421645

[22] Fotheringham AS, Brunsdon C, Charlton M. Geographically Weighted Regression: the Analysis of Spatially Varying Relationships. University of Newcastle: John Wiley & Sons; 2003.

[23] Wheeler D, Tiefelsdorf M. Multicollinearity and correlation among local regression coefficients in geographically weighted regression. J Geogr Syst. 2005;7(2):161–187.

[24] Wheeler DC, Calder CA. Bayesian spatially varying coefficient models in the presence of collinearity; 2006.

[25] Wheeler DC. Simultaneous coefficient penalization and model selection in geographically weighted regression: the geographically weighted lasso. Environ Plan. 2009;41(3):722–742.

[26] Czarnota J, Gennings C, Wheeler DC. Assessment of weighted quantile sum regression for modeling chemical mixtures and cancer risk. Cancer Inform. 2015;14:S17295. 10.4137/CIN.S17295 PMC4431483 26005323

[27] Shaddick G, Zidek JV. A case study in preferential sampling: long term monitoring of air pollution in the UK. Spat Stat. 2014;9:51–65.

[28] Diggle PJ, Tawn JA, Moyeed RA. Model-based Geostatistics. J. R. Stat. Soc. Ser. C 1998;47(3):299–350.

[29] Plummer M JAGS: A program for analysis of Bayesian graphical models using Gibbs sampling. Proceedings of the 3rd International Workshop on Distributed Statistical Computing; 2003:1–10.

[30] R Core Team. R: A Language and Environment for Statistical Computing. Vienna, Austria: R Foundation for Statistical Computing; 2021.

[31] Gelman A, Rubin DB. Inference from iterative simulation using multiple sequences. Stat Sci. 1992;7(4):457–472.

[32] Plummer M, Best N, Cowles K, Vines K. CODA: convergence diagnosis and output analysis for MCMC. R News. 2006;6:7–11. https://www.r-project.org/doc/Rnews/Rnews_2006-1.pdf

[33] Chatterjee N, Hartge P, Cerhan JR, Risk of non-Hodgkin’s lymphoma and family history of lymphatic, hematologic, and other cancers. Cancer Epidemiol Prev Biomarkers. 2004;13(9):1415–1421. 15342441

[34] Morton LM, Slager SL, Cerhan JR, Etiologic heterogeneity among non-Hodgkin lymphoma subtypes: the InterLymph non-Hodgkin lymphoma subtypes project. J Natl Cancer Inst Monogr. 2014;2014(48):130–144. 25174034 10.1093/jncimonographs/lgu013 PMC4155467

[35] ESRI. ArcView 3.2 GIS. Redlands: Environmental Systems Research Institute, Inc; (1999).

[36] Colt JS, Lubin J, Camann D, Comparison of pesticide levels in carpet dust and self-reported pest treatment practices in four US sites. J Expo Sci Environ Epidemiol. 2004;14(1):74–83. 10.1038/sj.jea.7500307 14726946

[37] Wheeler D, Czarnota J. Modeling Chemical Mixture Effects with Grouped Weighted Quantile Sum Regression. ISEE Conference Abstracts; 2016.

[38] Wheeler DC, Waller LA, Cozen W, Ward MH. Spatial-temporal analysis of non-Hodgkin lymphoma risk using multiple residential locations. Spat Spatiotemporal Epidemiol. 2012;3(2):163–171. 22682442 10.1016/j.sste.2012.04.009 PMC3372929

[39] Morton LM Etiologic heterogeneity among non-Hodgkin lymphoma subtypes. Blood. 2008;112(13):5150–5160. 18796628 10.1182/blood-2008-01-133587 PMC2597610

[40] IARC. Agents Classified by the IARC Monographs. International Agency for Research on Cancer; 2022 Accessed October 26, 2022. http://monographs.iarc.fr/ENG/Classification/index.php

[41] Hartge P Residential herbicide use and risk of non-Hodgkin lymphoma. Cancer Epidemiol. Biomarkers Prev. 2005;14(4):934–937. 15824166 10.1158/1055-9965.EPI-04-0730

[42] De Roos AJ Herbicide use in farming and other jobs in relation to non-Hodgkin’s lymphoma (NHL) risk. Occup Environ Med. 2022;79(12):795–806. 36207110 10.1136/oemed-2022-108371 PMC9669193

[43] Boyle J, Ward MH, Cerhan JR, Rothman N, Wheeler DC. Estimating mixture effects and cumulative spatial risk over time simultaneously using a Bayesian index low-rank kriging multiple membership model. Stat Med. 2022;41(29):5679–5697. 36161724 10.1002/sim.9587 PMC9691549

[44] Czarnota J, Wheeler DC, Gennings C. Evaluating geographically weighted regression models for environmental chemical risk analysis. Cancer Inform. 2015;14:S17296. 10.4137/CIN.S17296 PMC4426957 25983546

[45] Hodges JS, Reich BJ. Adding spatially-correlated errors can mess up the fixed effect you love. Am Stat. 2010;64(4):325–334.

