# Supplementary material for: Modeling variation in mixture effects over space with a Bayesian spatially varying mixture model
Source: Stat Med. Author manuscript; Available in PMC 2024 Mar 30. (PMC10964969; doi:10.1002/sim.10022)
Supplement: Supplemental_Material [file NIHMS1967742-supplement-Supplemental_Material.docx]

**Supplemental Material**

**Modeling variation in mixture effects over space with a Bayesian spatially varying mixture model**

Joseph Boyle^1^, Mary H. Ward^2^, James R. Cerhan^3^, Nat Rothman^2^, David C. Wheeler^1^

^1^ Department of Biostatistics, Virginia Commonwealth University, Richmond, VA

^2^ Occupational and Environmental Epidemiology Branch, Division of Cancer Epidemiology and Genetics, National Cancer Institute, Rockville, MD

^3^ Department of Quantitative Health Sciences, Mayo Clinic, Rochester, MN

| **Mixture** | **Detroit** | **Iowa** | **Los Angeles** | **Seattle** |
| --- | --- | --- | --- | --- |
| PCBs | | | | |
| Congener 105 | 5.64 [1.51, 18.35] | 4.16 [1.29, 12.46] | 3.89 [1.16, 10.43] | 3.70 [0.99, 14.14] |
| Congener 138 | 15.09 [3.39, 37.90] | 7.98 [2.56, 25.65] | 6.12 [2.11, 14.77] | 10.95 [2.10, 42.25] |
| Congener 153 | 21.10 [5.42, 51.60] | 9.69 [3.15, 30.40] | 7.58 [2.61, 19.46] | 14.74 [2.95, 49.70] |
| Congener 170 | 2.44 [0.57, 11.90] | 1.73 [0.42, 6.10] | 1.88 [0.43, 5.28] | 1.76 [0.34, 9.23] |
| Congener 180 | 6.88 [2.10, 24.20] | 5.90 [1.84, 18.40] | 5.07 [1.70, 13.12] | 7.32 [1.65, 28.53] |
| PAHs | | | | |
| Benz(a)anthracene | 749.00 [382.00, 1540.00] | 123.00 [62.00, 264.00] | 59.45 [39.48, 93.25] | 120.00 [69.95, 203.75] |
| Benzo(a)pyrene | 927.00 [479.00, 1730.00] | 126.00 [61.00, 286.50] | 70.95 [45.35, 123.00] | 134.50 [75.83, 241.75] |
| Benz(b)fluoranthene | 1650.00 [952.00, 3350.00] | 250.00 [132.50, 571.00] | 147.00 [87.93, 222.50] | 262.00 [149.25, 487.00] |
| Benz(k)fluoranthene | 581.00 [322.00, 1160.00] | 89.70 [43.55, 187.00] | 53.26 [30.16, 82.60] | 93.55 [55.13, 157.75] |
| Chrysene | 1220.00 [734.00, 2420.00] | 224.00 [134.50, 459.00] | 166.50 [106.75, 248.25] | 251.50 [145.00, 416.00] |
| Dibenz(ah)anthracene | 183.00 [101.00, 387.00] | 29.70 [11.04, 64.90] | 22.75 [9.75, 35.98] | 32.50 [19.46, 62.38] |
| Indeno(1,2,3-cd)pyrene | 1020.00 [521.00, 1980.00] | 132.00 [65.55, 301.50] | 93.45 [56.43, 147.00] | 152.00 [88.23, 279.50] |
| Pesticides I | | | | |
| Alpha-chlordane | 9.15 [2.41, 32.60] | 10.60 [2.82, 44.75] | 42.45 [8.91, 137.75] | 6.34 [1.74, 17.92] |
| Gamma-chlordane | 16.58 [3.44, 37.80] | 16.19 [3.92, 62.40] | 64.65 [21.88, 208.50] | 8.56 [2.23, 25.98] |
| Carbaryl | 45.97 [20.02, 125.00] | 49.79 [11.81, 350.50] | 56.50 [20.88, 270.25] | 37.62 [5.79, 207.01] |
| DDE | 19.02 [9.50, 47.90] | 24.40 [9.23, 52.75] | 26.65 [10.88, 53.30] | 12.12 [4.94, 25.93] |
| DDT | 64.64 [13.13, 274.00] | 129.00 [30.35, 399.50] | 100.00 [18.24, 285.50] | 49.50 [10.01, 161.00] |
| *O*-phenylphenol | 318.00 [169.00, 549.00] | 219.00 [146.00, 380.00] | 172.00 [101.50, 319.50] | 291.50 [154.75, 514.00] |
| Pentachlorophenol | 258.00 [106.00, 598.00] | 490.00 [243.50, 983.50] | 167.35 [83.35, 375.00] | 397.00 [188.00, 899.25] |
| Propoxur | 53.10 [17.25, 139.00] | 53.20 [16.85, 127.00] | 160.00 [59.25, 444.00] | 50.75 [21.22, 183.75] |
| Pesticides II | | | | |
| Chlorpyrifos | 87.40 [29.50, 454.00] | 62.70 [20.36, 228.00] | 212.50 [75.60, 707.75] | 53.30 [17.19, 209.00] |
| *Cis*-permethrin | 99.86 [20.71, 829.00] | 174.00 [29.40, 860.50] | 1200.00 [288.75, 4915.00] | 450.50 [77.95, 2835.00] |
| *Trans*-permethrin | 194.00 [13.54, 1490.00] | 374.00 [19.16, 1515.00] | 2275.00 [592.25, 9177.50] | 903.50 [132.36, 5452.50] |
| 2,4-D | 734.00 [245.00, 1680.00] | 1090.00 [345.00, 2950.00] | 75.81 [33.01, 179.75] | 273.50 [130.00, 1035.00] |
| Diazinon | 28.40 [4.49, 87.60] | 9.68 [2.30, 36.55] | 49.95 [11.37, 209.50] | 19.24 [3.15, 65.85] |
| Dicamba | 18.81 [4.88, 71.48] | 20.39 [5.32, 115.00] | 11.34 [2.74, 28.51] | 11.94 [2.83, 36.13] |
| Methoxychlor | 33.20 [8.99, 171.00] | 84.74 [13.64, 292.84] | 23.69 [6.37, 80.25] | 25.32 [5.80, 137.50] |

**Table S1.** Summary of chemical measurements in house dust by group and NCI-SEER study center.

**Note:** Quantities in table are median (1^st^ quartile, 3^rd^ quartile) for chemical measurements in ng per gram of dust.

**Table S2.** Summary of estimated chemical importance weights by group and NCI-SEER study center.

| **Mixture** | **Detroit** | **Iowa** | **Los Angeles** | **Seattle** |
| --- | --- | --- | --- | --- |
| PCBs | | | | |
| Congener 105 | 0.210 | 0.186 | 0.193 | 0.193 |
| Congener 138 | 0.189 | 0.204 | 0.205 | 0.198 |
| Congener 153 | 0.188 | 0.212 | 0.209 | 0.202 |
| Congener 170 | 0.211 | 0.193 | 0.203 | 0.200 |
| Congener 180 | 0.202 | 0.205 | 0.190 | 0.207 |
| PAHs | | | | |
| Benz(a)anthracene | 0.143 | 0.150 | 0.136 | 0.144 |
| Benzo(a)pyrene | 0.139 | 0.139 | 0.133 | 0.140 |
| Benz(b)fluoranthene | 0.143 | 0.137 | 0.148 | 0.135 |
| Benz(k)fluoranthene | 0.142 | 0.144 | 0.158 | 0.147 |
| Chrysene | 0.146 | 0.149 | 0.141 | 0.144 |
| Dibenz(ah)anthracene | 0.144 | 0.141 | 0.149 | 0.143 |
| Indeno(1,2,3-cd)pyrene | 0.143 | 0.140 | 0.135 | 0.147 |
| Pesticides I | | | | |
| Alpha-chlordane | 0.111 | 0.107 | 0.122 | 0.124 |
| Gamma-chlordane | 0.120 | 0.108 | 0.127 | 0.118 |
| Carbaryl | 0.107 | 0.084 | 0.122 | 0.120 |
| DDE | 0.127 | 0.169 | 0.128 | 0.126 |
| DDT | 0.128 | 0.124 | 0.129 | 0.115 |
| *O*-phenylphenol | 0.141 | 0.128 | 0.117 | 0.117 |
| Pentachlorophenol | 0.142 | 0.113 | 0.120 | 0.144 |
| Propoxur | 0.124 | 0.167 | 0.135 | 0.136 |
| Pesticides II | | | | |
| Chlorpyrifos | 0.127 | 0.079 | 0.139 | 0.141 |
| *Cis*-permethrin | 0.163 | 0.080 | 0.132 | 0.123 |
| *Trans*-permethrin | 0.153 | 0.091 | 0.139 | 0.124 |
| 2,4-D | 0.133 | 0.390 | 0.141 | 0.151 |
| Diazinon | 0.134 | 0.117 | 0.151 | 0.138 |
| Dicamba | 0.142 | 0.161 | 0.142 | 0.171 |
| Methoxychlor | 0.148 | 0.082 | 0.156 | 0.152 |

**Figure S1.** Average estimated coefficient surface for simulation study scenario 2A and constant effect.


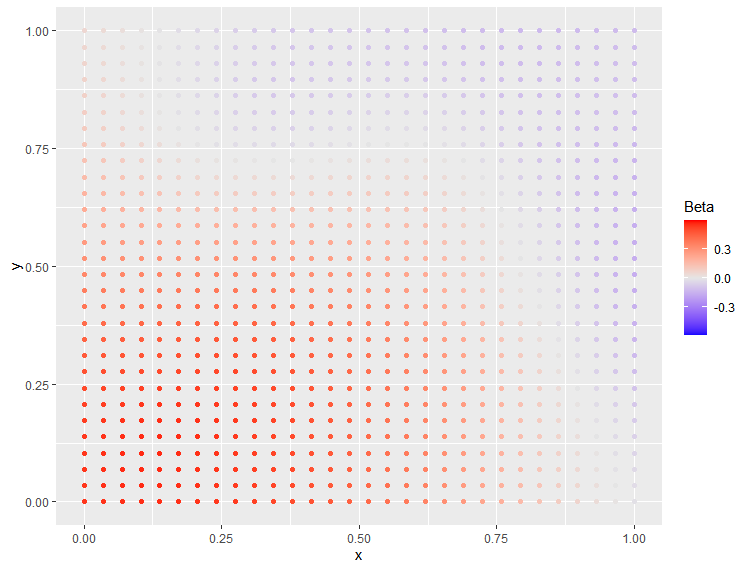


**Figure S2.** Average estimated coefficient surface for simulation study scenario 2B and constant effect.


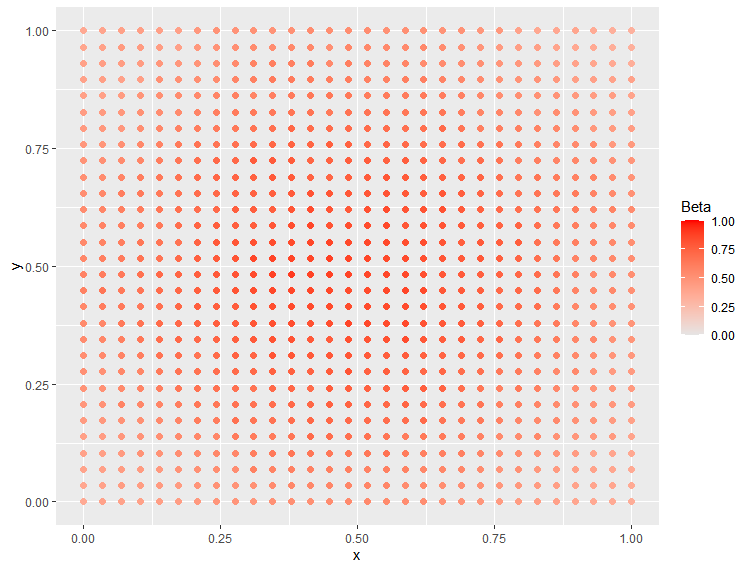


**Figure S3.** Significant Pesticides I mixture effects in Iowa, with positive significant effects shown in red.


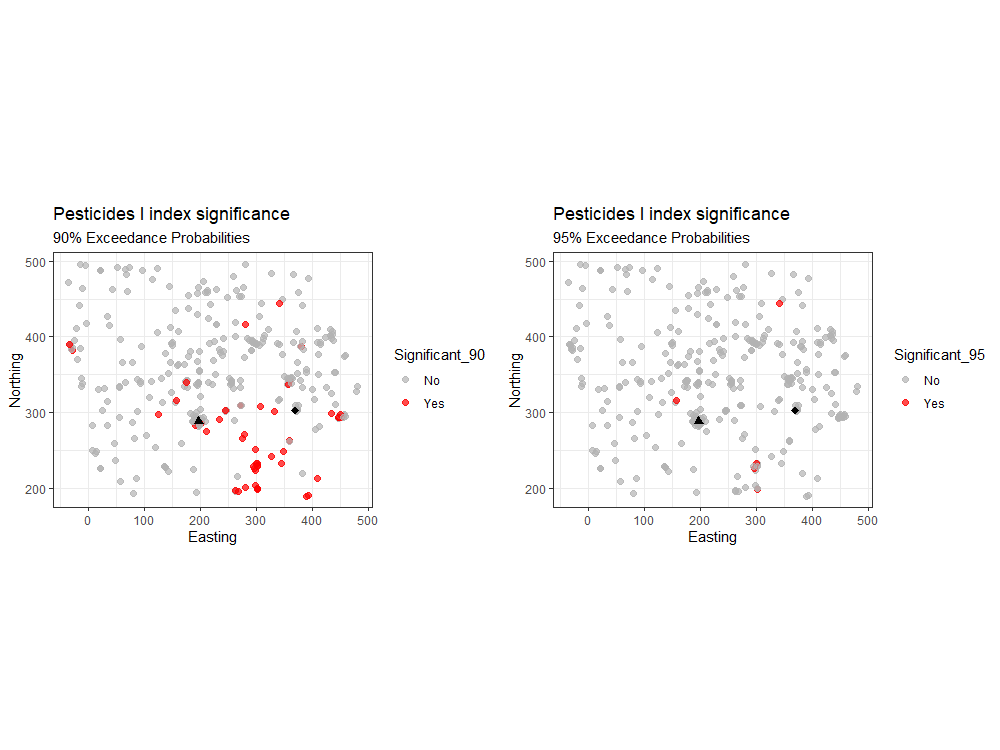


**Note**: Des Moines and Iowa City displayed with black triangle and diamond, respectively.


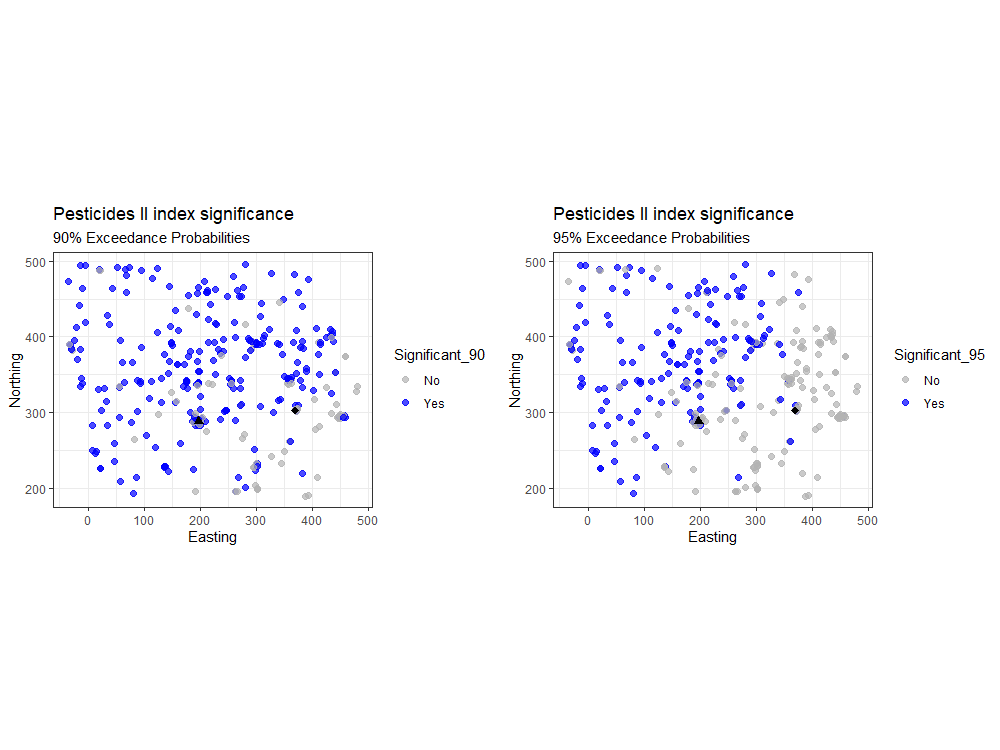
**Figure S4.** Significant Pesticides II mixture effects in Iowa, with negative significant effects shown in blue.

**Note**: Des Moines and Iowa City displayed with black triangle and diamond, respectively.

**Appendix S1. Derivation of posterior distribution in Bayesian spatially varying mixture model**

We provide the derivation of the posterior distribution of spatially varying mixture effects $\beta$ in the model in the case where there is one spatially-varying intercept $\beta_{1i}$, one spatially-varying mixture coefficient $\beta_{2i}$ applied to chemical mixture index $x_{i}$, and the response variable $Y_{i}$ is dichotomous, as in a case-control study. For simplicity, we omit adjustment covariates.

For each vector of regression coefficients $\boldsymbol{\beta}_{\boldsymbol{k}},k=1,2,$ we place multivariate normal priors with mean vector $\boldsymbol{0}$ and covariance matrix of $\boldsymbol{\Sigma}_{\boldsymbol{k}}$. For $\boldsymbol{\Sigma}_{\boldsymbol{k}}$, we assume a Matern family for the covariance between mixture effect values over space, fixing parameters $m=1$ and $\nu=\frac{3}{2}$ and estimating the range parameter $\rho_{k}$ for each spatially-varying coefficient, $k=1,2$. Thus, the $\left( i,j \right)^{th}$ entry of the covariance matrix $\boldsymbol{\Sigma}_{\boldsymbol{k}},k=1,2$ is given by

$\left( 1+d\left( i,j \right)/\rho_{k} \right)\exp\left( -d\left( i,j \right)/\rho_{k} \right)$,

where $d\left( i,j \right)$ represents the distance between the $i^{th}$ and $j^{th}$ participants. Finally, we assign noninformative uniform distributions to $\rho_{k}$ taken over the range of inter-point distances.

Defining $\phi\left( \cdot\right)=invlogit\left( \cdot\right)=\frac{\exp\left( \cdot\right)}{1+\exp\left( \cdot\right)}$, by Bayes Rule, the joint posterior distribution of $\beta$ is then

$$\boldsymbol{p}\left( \boldsymbol{\beta} | \boldsymbol{Y} \right)\boldsymbol{\propto}\boldsymbol{p}\left( \boldsymbol{Y} | \boldsymbol{\beta} \right)\boldsymbol{p}\left( \boldsymbol{\beta} \right)$$

$$\boldsymbol{\propto}\boldsymbol{[}\prod_{\boldsymbol{i=1}}^{\boldsymbol{n}} \left( \boldsymbol{\phi}\left( \boldsymbol{X}_{\boldsymbol{i}}\boldsymbol{\beta} \right) \right)^{\boldsymbol{y}_{\boldsymbol{i}}}\left( \boldsymbol{1-}\boldsymbol{\phi}\left( \boldsymbol{X}_{\boldsymbol{i}}\boldsymbol{\beta} \right) \right)^{\boldsymbol{1-}\boldsymbol{y}_{\boldsymbol{i}}}\boldsymbol{]}\boldsymbol{*}\boldsymbol{[}\prod_{\boldsymbol{k=1}}^{\boldsymbol{2}} \left( \boldsymbol{2}\boldsymbol{\pi} \right)^{\boldsymbol{-n}\boldsymbol{/}\boldsymbol{2}}det\left( \boldsymbol{\Sigma}_{\boldsymbol{k}} \right)^{\boldsymbol{-1/2}}exp \left( \boldsymbol{-}\frac{\boldsymbol{1}}{\boldsymbol{2}}\left( \boldsymbol{\beta}_{\boldsymbol{k}}\boldsymbol{-}\boldsymbol{\mu}_{\boldsymbol{k}} \right)^{\boldsymbol{T}}\boldsymbol{\Sigma}_{\boldsymbol{k}}^{-1}\left( \boldsymbol{\beta}_{\boldsymbol{k}}\boldsymbol{-}\boldsymbol{\mu}_{\boldsymbol{k}} \right) \right)\boldsymbol{]}$$

Then, the conditional posterior of the spatially-varying intercept can be simplified to

$$p\left( \boldsymbol{\beta}_{\boldsymbol{1}} | \boldsymbol{Y} \right)\propto\left[ \prod_{i:Y_{i}=1} \phi\left( \boldsymbol{X}_{\boldsymbol{i}}\boldsymbol{\beta} \right) \right]\left[ \prod_{i:Y_{i}=0} \left( 1-\phi\left( \boldsymbol{X}_{\boldsymbol{i}}\boldsymbol{\beta} \right) \right) \right]det\left( \boldsymbol{\Sigma}_{\boldsymbol{1}} \right)^{-1/2}\exp\left( -\frac{1}{2}\boldsymbol{\beta}_{\boldsymbol{1}}^{\boldsymbol{T}}\boldsymbol{\Sigma}_{\boldsymbol{1}}^{-1}\boldsymbol{\beta}_{\boldsymbol{1}} \right)$$

The conditional posterior of the spatially-varying mixture effect can be simplified to

$$p\left( \boldsymbol{\beta}_{2} | \boldsymbol{Y} \right)\propto\left[ \prod_{i:Y_{i}=1} \phi\left( \boldsymbol{X}_{\boldsymbol{i}}\boldsymbol{\beta} \right) \right]\left[ \prod_{i:Y_{i}=0} \left( 1-\phi\left( \boldsymbol{X}_{\boldsymbol{i}}\boldsymbol{\beta} \right) \right) \right]det\left( \boldsymbol{\Sigma}_{\boldsymbol{2}} \right)^{-1/2} exp\left( -\frac{1}{2}\boldsymbol{\beta}_{\boldsymbol{2}}^{\boldsymbol{T}}\boldsymbol{\Sigma}_{\boldsymbol{2}}^{-1}\boldsymbol{\beta}_{\boldsymbol{2}} \right)$$

Parameter estimation proceeds with Gibbs sampling using a suitable burn-in period.

**Appendix S2. Model code in JAGS**

### Load libraries and read in data
library(tidyverse); library(rjags); library(runjags); library(coda)
df <- read.csv("data_1.csv"); n <- nrow(df)
### Extract chemical mixture component
chemicals_mat <- as.matrix(as.data.frame(df[,5:10)); J <- ncol(chemicals_mat)
## Calculate distance between participants for regression coefficients
bb_dist <- matrix(0, nrow = nrow(df), ncol = nrow(df))
for (i in 1:nrow(df)){
 for (j in 1:nrow(df)){
 bb_dist[i,j] <- sqrt((df$x[i] - df$x[j])^2 + (df$y[i] - df$y[j])^2)
 }
}
rho_max <- max(c(bb_dist))

### Datalist ###
datalist <- list('n' = nrow(df), 'case' = df$case, 'bb_dist' = bb_dist,
 'rho_max' = rho_max, 'chem' = chemicals_mat, 'J' = ncol(chemicals_mat))

### MODEL ###
modelfile <- ' model {
for (i in 1:n){
 case[i] ~ dbern(p[i])
 logit(p[i]) <- beta1[i] + beta2[i]*(sum(chem[i, 1:J] %*% pi1[1:J]))
}

####### Priors
beta1[1:n] ~ dmnorm(rep(0,n), tau_s1 * inverse(bb_mat1))
tau_s1 <- 1/(sigma_s1*sigma_s1)
sigma_s1 ~ dunif(0,100)

beta2[1:n] ~ dmnorm(rep(0,n), tau_s2 * inverse(bb_mat2))
tau_s2 <- 1/(sigma_s2*sigma_s2)
sigma_s2 ~ dunif(0,100)

for (i in 1:n){
 for (j in 1:n){
 bb_mat1[i,j] <- (1 + abs(bb_dist[i,j] / rho1)) * exp(-abs(bb_dist[i,j] / rho1))
 bb_mat2[i,j] <- (1 + abs(bb_dist[i,j] / rho2)) * exp(-abs(bb_dist[i,j] / rho2))
 }
}
rho1 ~ dunif(0, rho_max)
rho2~ dunif(0, rho_max)

for (j in 1:J){
 pi[j] <- edelta[j] / sum(edelta[1:J])
 edelta[j] <- exp(delta[j])
 delta[j] ~ dnorm(0, tau_i)
}
tau_i <- 1/(sigma_i*sigma_i)
sigma_i ~ dunif(0,10)
}
'

We note that it is also possible to adjust for covariates, to fit a global (not spatially-varying) intercept, and/or to fix fewer parameters in the Matern covariance function (i.e., estimate more parameters) in this model. But the latter choice may lead to convergence issues, and we have found that fixing $m$ and $\nu$ and estimating $\rho$ often leads to good performance.
